# Cortical Auditory Evoked Potentials in Response to Frequency Changes with Varied Magnitude, Rate, and Direction

**DOI:** 10.1007/s10162-019-00726-2

**Published:** 2019-06-05

**Authors:** Bernard M.D. Vonck, Marc J.W. Lammers, Marjolijn van der Waals, Gijsbert A. van Zanten, Huib Versnel

**Affiliations:** 1 Department of Otorhinolaryngology and Head & Neck Surgery, University Medical Center Utrecht, Room G.02.531, P.O. Box 85500, 3508 GA Utrecht, The Netherlands; 2 UMC Utrecht Brain Center, Utrecht, The Netherlands; 3 BC Rotary Hearing and Balance Centre at St. Paul’s Hospital, University of British Columbia, Vancouver, British Columbia Canada

**Keywords:** acoustic change complex, cortical auditory evoked potential, frequency modulation, electroencephalography

## Abstract

Recent literature on cortical auditory evoked potentials has focused on correlations with hearing performance with the aim to develop an objective clinical tool. However, cortical responses depend on the type of stimulus and choice of stimulus parameters. This study investigates cortical auditory evoked potentials to sound changes, so-called acoustic change complexes (ACC), and the effects of varying three stimulus parameters. In twelve normal-hearing subjects, ACC waveforms were evoked by presenting frequency changes with varying magnitude, rate, and direction. The N1 amplitude and latency were strongly affected by magnitude, which is known from the literature. Importantly, both of these N1 variables were also significantly affected by both rate and direction of the frequency change. Larger and earlier N1 peaks were evoked by increasing the magnitude and rate of the frequency change and with downward rather than upward direction of the frequency change. The P2 amplitude increased with magnitude and depended, to a lesser extent, on rate of the frequency change while direction had no effect on this peak. The N1–P2 interval was not affected by any of the stimulus parameters. In conclusion, the ACC is most strongly affected by magnitude and also substantially by rate and direction of the change. These stimulus dependencies should be considered in choosing stimuli for ACCs as objective clinical measure of hearing performance.

## Introduction

The ability of our auditory system to detect modulations within ongoing sounds is essential for daily life. It enables us to detect changes in our environment, to identify vowels and consonants, and also to appreciate pitch differences in musical compositions. Normal hearing listeners can identify very small frequency changes of less than 1 % of the base frequency (Sek and Moore 1995; Amitay et al. 2006; Papakonstantinou et al. 2011). In the case of sensorineural hearing loss, frequency resolution capabilities become impaired, resulting in poorer speech understanding in especially noisy environments (Dreschler and Plomp 1985; Horst 1987; Noordhoek et al. 2001; Strelcyk and Dau 2009; Papakonstantinou et al. 2011). Psychophysical experiments revealed that patients with sensorineural hearing loss had frequency discrimination thresholds which correlated well with their speech perception in noise abilities (Noordhoek et al. 2001; Papakonstantinou et al. 2011). These findings indicate the importance of frequency discrimination in oral communication.

The underlying neurophysiological alterations in response to frequency changes have been investigated using cortical auditory evoked potentials (Arlinger et al. 1976; McCandless and Rose 1970; Dimitrijevic et al. 2008; Harris et al. 2008; Pratt et al. 2009). The obligatory cortical auditory evoked potential is thought to mainly reflect neuronal activation in various primary and secondary auditory cortical fields (Eggermont and Ponton 2002; Martin et al. 2007, 2008). The response evoked by a change in a continuous stimulus is often called the acoustic change complex (ACC). This response can be elicited in response to changes within speech stimuli (Ostroff et al. 1998; Martin and Boothroyd 2000; Tremblay et al. 2003; Friesen and Tremblay 2006) and to intensity or frequency changes within continuous tones (McCandless and Rose 1970; Arlinger et al. 1976; Harris et al. 2007, 2008; Dimitrijevic et al. 2008; Pratt et al. 2009; Presacco and Middlebrooks 2018).

Recent literature has shown interest in the ACC and investigated its possible clinical applications (He et al. 2012; Brown et al. 2015, 2017; Chen and Small 2015; Kim 2015). The ACC has been reported to correlate with psychophysical measures in normal hearing adult subjects (He et al. 2012; Brown et al. 2017), and moreover, the ACC has been reliably evoked in various types of subjects such as young children, hearing aid users, cochlear implant users, and sedated cats (Tremblay et al. 2006; He et al. 2012; Brown et al. 2015; Chen and Small 2015; Presacco and Middlebrooks 2018). The ACC might therefore possess characteristics, which can be used for objective auditory assessment. Although recent research has aimed at the correlation of ACC measures (such as amplitude and latency) to psychophysical outcomes, these measures also depend on the choice of stimulus parameters. Knowledge on how different factors of frequency change stimuli affect ACC parameters is therefore essential for researchers investigating the ACC. Previous studies have revealed that ACCs elicited with frequency changes show larger amplitudes with increasing magnitude of the frequency change (McCandless and Rose 1970; Martin and Boothroyd 2000; Harris et al. 2008; Pratt et al. 2009; He et al. 2012). Harris et al. (2008) demonstrated that ACCs can even be evoked in response to small changes of less than 1 % of the base frequency, closely resembling behavioral just noticeable frequency discrimination results. Besides the magnitude of the change, there are multiple parameters to choose for an acoustic change stimulus, see for instance, the differences in stimuli between abovementioned studies. The majority of studies using a pure tone stimulus, followed by a frequency change, studied only one direction of frequency change (frequency increase or decrease). With respect to rate of the frequency change, some authors did not report rates (Pratt et al. 2009; Brown et al. 2017) while others reported a constant duration of the change with varying magnitudes, thus resulting in varying velocities (Dimitrijevic et al. 2008; Harris et al. 2008; Presacco and Middlebrooks 2018). Thus, since evidence is lacking on the effect of rate and direction, we aim to investigate the extent to which suprathreshold ACC is influenced by rate and direction, next to magnitude. This may help researchers and clinicians to determine stimulus parameters when recording ACCs, for example, the rate and/or direction of change that generates the clearest response or the steepest amplitude–change slope. This may attribute to development of the ACC into a clinically applicable objective measurement of auditory performance. Therefore, in the current study, we systematically varied magnitude, rate, and direction to elicit the ACC in young, normal-hearing subjects.

## Methods

### Subjects

Twelve healthy, normal hearing volunteers, aged between 18 and 30 years old, agreed to participate in this study. Eleven participants were right handed, and one was left handed. Harris et al. (2008) reported that in a group of normal hearing subjects of this age, small frequency changes up to the threshold of around 0.8–1.8 % frequency change of the base frequency elicited a cortical response. In older adults, aged 65–80 years, this threshold ranged from 1.2 to 3.4 % of the base frequency (Harris et al. 2008). This study only included young subjects to diminish the possible effect of age on the ACC. Hearing thresholds of each participant measured by standard pure tone audiometry prior to the experiments were < 20 dB HL at each frequency (0.25–8 kHz). None of the subjects reported a history of hearing loss or tinnitus.

### Stimuli and Recording Procedure

**Fig. 1:**
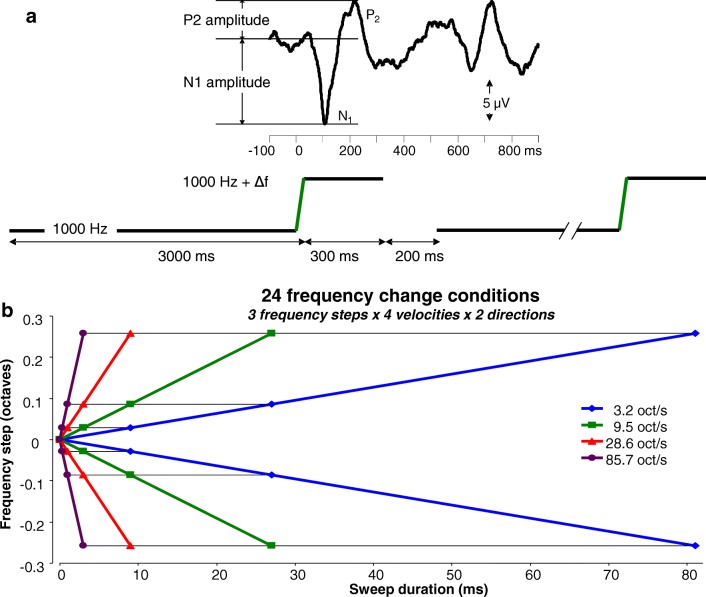
**a** Schematic presentation of the stimulus. Stimuli consisted of 3 parts: (1) a 1000-Hz pure tone with a duration of approximately 3 s, which transfers after 2919 to 2999.7 ms into (2) an upward or downward frequency modulated sweep with a duration of 0.33 to 81 ms and then proceeds as a (3) 300-ms continuous tone with a frequency of 1000 Hz ± Δ*f*. An inter stimulus interval of 200 ms was provided before the onset of the next repeated stimulus. The top graph demonstrates the corresponding acoustic change complex occurring at approximately 100 ms after the sweep and the cortical auditory evoked response at approximately 600 ms elicited by the onset of the new stimulus. **b** Schematic overview of all 24 stimulus conditions showing frequency step magnitude as a function of duration of the FM sweep. Downward frequency changes are indicated with negative values. Purple lines indicate the fastest sweeps of 85 oct/s, the red line sweeps of 28 oct/s, the green lines of 9.5 oct/s, and the blue lines of 3.2 oct/s. For each rate, frequency changes of 0.028, 0.086, and 0.257 oct in both upward and downward directions were used. The 24 frequency changes had varying durations: 0.33, 1, 3, 9, 27, or 81 ms.

Acoustic change complexes were evoked using tonal stimuli of 3300 ms, consisting of three components (Fig. 1a): (a) a reference tone of 1000 Hz with a duration of approximately 3 s, (b) a logarithmic frequency modulation (FM) sweep with a frequency change Δ*f*, and (c) a 300-ms target tone with a frequency 1000 − Δ*f* or 1000 + Δ*f* Hz. The FM sweep was generated using the following equation:

$$ y=\sin \left[\frac{2\pi {f}_0}{R\log (2)}{e}^{Rt\log (2)}-\varphi .\right] $$

with *y*: FM sweep signal; *f*_0_: reference frequency in Hz; *R*: rate in octave per second; *t*: time in seconds; and *φ*: phase correction to start FM sweep at the final phase of the reference tone.

The silent interval between stimuli was 200 ms. The choice of the relatively long duration of the reference tone was based on pilot data we obtained in 3 subjects, in which ACC amplitudes following a 3-s reference tone were substantially larger (by a factor ~ 1.5) than those following a 1-s reference tone. Time 0 of the recordings is defined by the onset of the frequency change.

Frequency changes of 3 different magnitudes were used: 0.028 oct (20 Hz for up change), 0.086, and 0.257 oct. The smallest magnitude of 0.028 oct corresponds to a 2 % frequency change from the reference tone, which is expected to elicit an ACC since it is above reported thresholds (0.8–1.8 %) in young normal-hearing individuals (Harris et al. 2008). These 3 magnitudes were presented at 4 different FM sweep velocities, 3.2, 9.5, 28, and 85 oct/s, which generated 12 stimulus conditions. These 12 frequency changes were presented in both the upward (+ Δ*f*) and downward (− Δ*f*) directions, resulting in a total of 24 different frequency change conditions for each participant (Fig. 1b). According to the combinations of steps and velocities, the 24 frequency changes had durations of 0.33, 1, 3, 9, 27, or 81 ms, as illustrated in Fig. 1b. The exact duration of the reference tone was 3000 ms minus the duration of the change and thus varied between 2919 and 2999.7 ms. Sound stimuli were generated using MATLAB (version 7.11.0, Mathworks, Natick, MA, USA) at a sample frequency of 50 kHz and presented monaurally to the left ear through a TDH-39 headphone at a level of 75 dB SPL. All 24 conditions were presented in a random order for each participant.

Participants were seated in a comfortable reclining chair in an electrically shielded, sound attenuated booth, and were allowed to watch a silent, captioned movie. They were carefully instructed prior to each recording to minimize movements and to fixate on the center of the video screen to minimize muscle and eye movement artifacts. Electrophysiological responses were recorded by Ag/AgCl electrodes placed according to the 10–20 system using a Medelec Synergy T-10 Evoked Potential system. The active electrode was placed at the vertex of the skull, Cz; the contralateral mastoid (right) was used as reference electrode; and the ground electrode was placed on the forehead. Eye movements and blinks were monitored using electrodes above and below the eye, contralateral of the stimulated ear. Electrode impedances were kept below 5 kΩ. The electrode signals were filtered from 0.01 to 100 Hz and recorded with a sampling rate of 50 kHz. Responses were acquired in a 1000-ms time window, including a prestimulus period of 100 ms. Responses containing amplitudes of > 100 μV at any electrode were rejected and excluded from the averaged response. For each condition, 100 accepted sweeps were averaged.

### Data Analyses

Averaged evoked potential data were used for determining peak amplitudes and latencies for each subject. The first peak, P1, was considerably smaller compared to the following N1 and P2 peaks. The low signal-to-noise ratio of this peak impedes reliable determination of P1 amplitude and latency. Therefore, only the N1 and P2 were further analyzed. The N1 of the ACC was defined as the most negative peak at 70 to 170 ms after the onset of the frequency change. P2 was defined as the first pronounced positive peak occurring after N1 at 150 to 250 ms after the change. The N1 and P2 amplitudes were computed relative to baseline, which was assessed by the average prestimulus segment (− 100 to 0 ms; see Fig. 1a). The N1 latency was measured at the peak relative to stimulus onset, and the P2 latency was analyzed relative to the N1 latency. Statistical analyses were completed using SPSS version 22.0 software (IBM, Armonk, NY, USA). For each of the 24 conditions, amplitudes and latencies of both the N1 and P2 peaks were averaged across all 12 subjects. Repeated measures (rm) ANOVA was applied to evaluate differences between stimulus conditions with respect to the three within-subject factors magnitude (3 levels), rate (4 levels), and direction (2 levels). If the assumption of sphericity was violated, the Greenhouse–Geisser correction was applied; *p* < 0.05 was considered significant for main effects and interactions. In a secondary evaluation, an effect of direction was analyzed by computing a direction selectivity index for amplitude: DSI = (Amplitude down − Amplitude up) / (Amplitude down + Amplitude up), as described e.g. by Nelken and Versnel (2000), and by computing latency differences between directions. DSI and latency differences were statistically tested using Bonferroni corrected *t* tests.

## Results

**Fig. 2:**
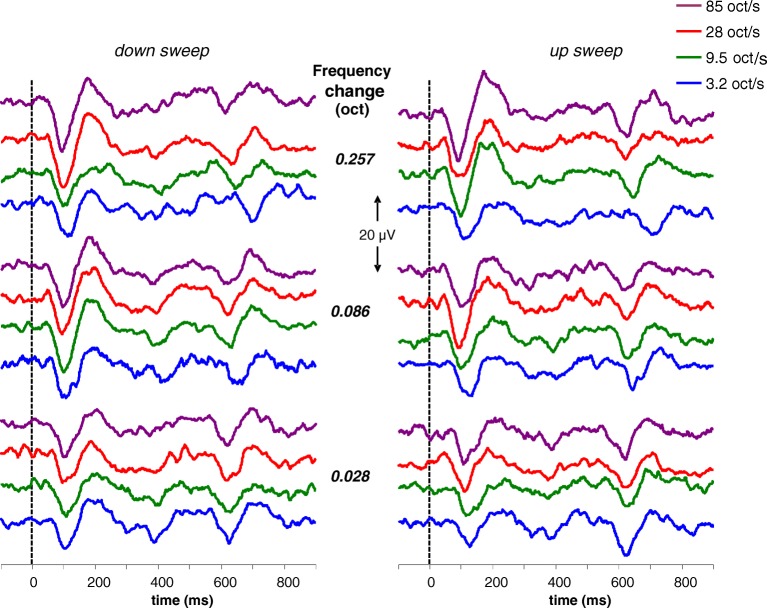
Individual and unfiltered recordings obtained in one subject displaying the acoustic change responses for all 24 conditions separately. The frequency change starts at 0 ms, and the acoustic change complex can be identified in all traces at approximately 100 ms after the frequency change. The response occurring around 600 ms after the frequency change is the cortical onset response on the next repeated stimulus. The eight top traces demonstrate the ACCs obtained with the largest frequency change, i.e., of 0.257 oct; the middle traces the responses for a frequency change of 0.086 oct; and the bottom traces for a change of 0.028 oct. Purple traces indicate the ACCs for the fastest sweeps, i.e., of 85 oct/s, the red traces for 28 oct/s, the green traces for 9.5 oct/s, and the blue traces for 3.2 oct/s. The traces on the left show ACCs for downward and the traces on the right for upward frequency changes

Reproducible and clear ACC responses exhibiting the typical N1–P2 waveform morphology were evoked in all 12 subjects, for all 24 stimulus conditions. This is illustrated by Fig. 2, which shows the unfiltered responses evoked in one individual subject for the 24 different conditions. ACC responses with an N1 peak at approximately 100 ms are seen for all stimulus conditions. Around 600 ms, a smaller N1–P2 waveform occurs elicited by the onset of the next repeated stimulus, which starts with the 1000 Hz reference tone prior to the frequency change as illustrated in Fig. 1a. In some recordings, a small N1–P2 can be identified at approximately 400 ms, elicited in response to the offset of the stimulus.

### Effects of Frequency Change Magnitude, Rate, and Direction on Amplitude

**Fig. 3:**
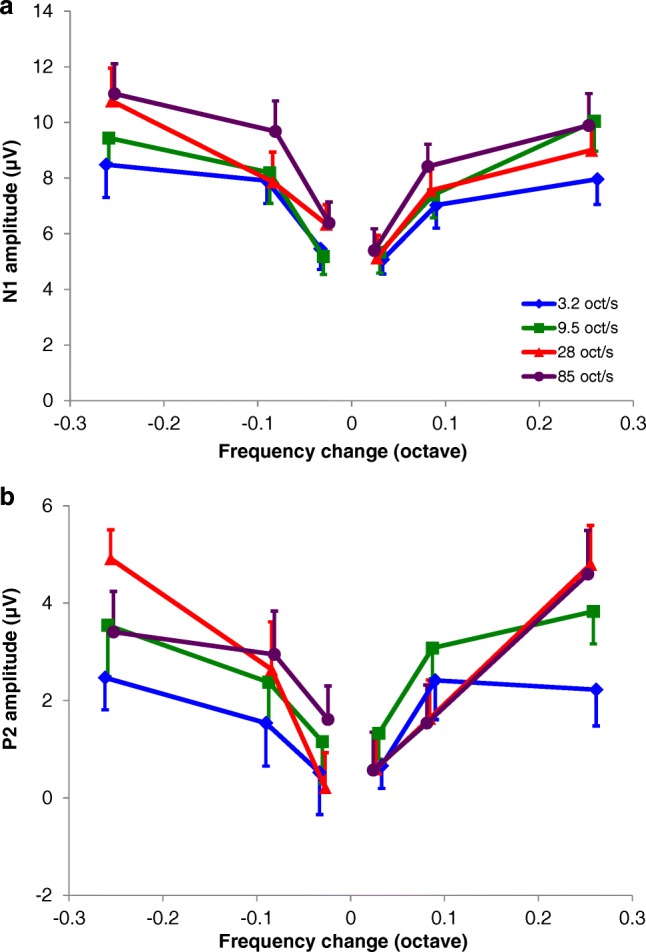
Average N1 (**a**) and P2 (**b**) amplitudes as a function of magnitude of the frequency change (*n* = 12). Downward frequency changes are presented on the left (indicated with negative values) and upward changes on the right. Purple lines indicate the sweeps of 85 oct/s, red lines of 28 oct/s, green lines of 9.5 oct/s, and the blue lines of 3.2 oct/s. Error bars indicate standard errors of the mean

As is seen in the examples presented in Fig. 2, the ACC amplitude increased with increasing magnitude of the frequency change. In addition, the ACC amplitude increased with increasing rate. This effect can be well observed within the top traces of Fig. 2 (ACCs evoked by the largest frequency change of 0.257 oct). With respect to direction, there appears to be little difference in ACC amplitude between downward (left traces) and upward (right traces) sweeps in this subject. Figure 3a depicts the averaged amplitudes of all subjects (*N* = 12) as a function of frequency change magnitude and shows that the N1 amplitude of the ACC was significantly affected by magnitude, rate, and direction of the frequency step. Amplitudes increased with increasing magnitude of the frequency changes (rm ANOVA; *F*(2, 22) = 50.0, *p* < 0.0001). The largest increase in amplitude occurred with the increase from 0.028 to 0.086 oct. Furthermore, the N1 amplitude increased with an increase in rate (rm ANOVA; *F*(3, 33) = 11.1, *p* < 0.0001). Amplitudes evoked by downward changes in frequency were larger compared to amplitudes evoked by upward changes (rm ANOVA; *F*(1,11) = 9.9, *p* = 0.009). Interaction effects were revealed between magnitude and rate (rm ANOVA; magnitude × rate *F*(3,33) = 3.1, *p* = 0.038) and between rate and direction (rm ANOVA; rate × direction *F*(6,66) = 3.1, *p* = 0.011). Figure 3 illustrates these effects: a small amplitude difference between velocities at the smallest frequency change in contrast to a larger difference at the largest frequency change and a larger rate effect for downward than for upward changes.

As with the N1 component, the P2 increased with magnitude (rm ANOVA; *F*(2, 22) = 30.2, *p* < 0.0001) and, to a lesser extent, with rate of the frequency change (rm ANOVA *F*(3, 33) = 3.1, *p* = 0.039) (Fig. 3b). In contrast to the N1 peak, P2 amplitude was not affected by direction. An interaction effect for the P2 amplitude was revealed between magnitude and rate of the frequency change (rm ANOVA; magnitude × rate *F*(6,66) = 3.5, *p* = 0.005). This effect was reflected by a larger increase in amplitudes for higher than for lower velocities, in especially the upward sweeps (Fig. 3b). With upward frequency changes of 0.028 and 0.086 oct, the slower sweeps showed larger amplitudes while with a frequency change of 0.257 the faster sweeps showed larger amplitudes. P2 amplitude was not affected by direction of the change (rm ANOVA; *F*(2, 22) = 0.01, *p* = 0.924).

**Fig. 4:**
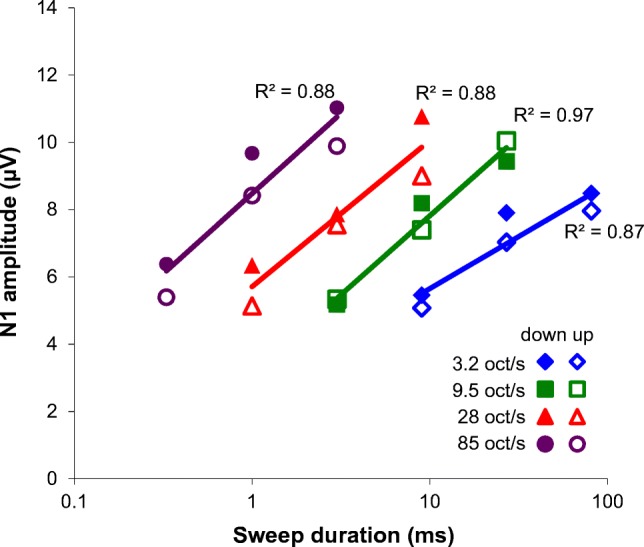
N1 amplitude as a function of sweep duration, presented for each rate separately and averaged across all 12 subjects. The purple line indicates the fastest sweeps of 85 oct/s, the red line indicates 28 oct/s, the green line 9.5 oct/s, and the blue line 3.2 oct/s. Downward sweeps are indicated with solid symbols and upward sweeps with open symbols. The increase of N1 amplitude (*A*), with frequency change (Δ*f*), can be described as follows: *A* ~ *b* log(Δ*f*). The slope *b* for a rate of 85 oct/s is 4.8, for 28 oct/s 4.3, for 9.5 oct/s 4.7, and for 3.2 oct/s 3.1

Figure 4 shows the N1 amplitude as function of the logarithm of the sweep duration for each of the four rates. Considering the goodness of the fit (*R*^2^ > 0.8), it appears that the increase of the N1 amplitude, at least in the range applied here, can be described as a logarithmic function of the sweep duration. The slope varied from 3.1 μV for a rate of 3.2 oct/s to 4.8 μV for a rate of 85 oct/s. The shallower slope for slower changes reflects the interaction between magnitude and rate. Since the magnitude of change is proportional to the sweep duration (magnitude = rate × duration), the N1 amplitude increases logarithmically with the magnitude.

### Effects of Frequency Change Magnitude, Rate, and Direction on Latency

**Fig. 5:**
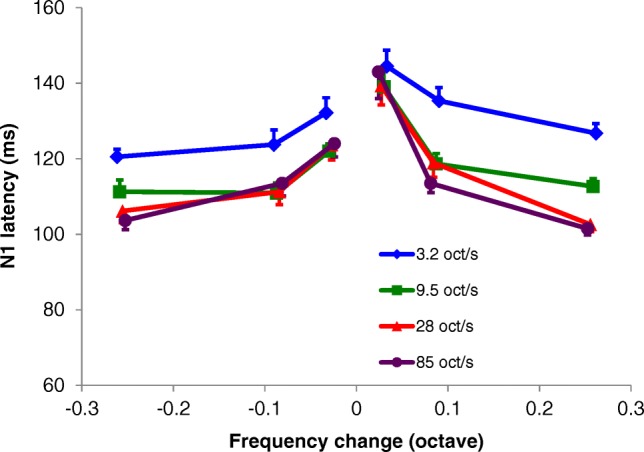
Average N1 latency as a function of magnitude of the frequency change (*n* = 12). Downward frequency changes are presented on the left (indicated with negative values) and upward changes on the right. Purple lines indicate the sweeps of 85 oct/s, red lines of 28 oct/s, green lines of 9.5 oct/s, and blue lines of 3.2 oct/s. Error bars indicate standard errors of the mean

The N1 latency was significantly affected by magnitude, rate and direction of the frequency change (Fig. 5). Latencies decreased with an increase in magnitude of the frequency change (rm ANOVA; *F*(2, 22) = 86.2, *p* < 0.0001) and as with the N1 amplitude, this effect was strongest with the increase from 0.028 to 0.086 oct. Furthermore, the latency was affected by rate of the change (*F*(3, 33) = 40.6, *p* < 0.0001), i.e., latency decreased with increasing rate. In particular, the slowest rate of 3.2 oct/s produced substantially longer N1 latencies than the other velocities, by approximately 10 ms, over all frequency changes (Fig. 5). In addition, for the largest frequency change of 0.257 oct, the N1 latency was longer for both the slowest velocities compared to the two highest velocities. This was confirmed by interaction effects between magnitude and rate of the frequency change (rm ANOVA; magnitude × rate *F*(6,66) = 6.45, *p* < 0.0001) and between direction, magnitude, and rate of the frequency change (rm ANOVA; direction × magnitude × rate *F*(6,66) = 2.41, *p* = 0.036). As shown in Fig. 5, the average N1 latencies per stimulus condition were shorter when evoked in response to downward changes (range of averages 104–132 ms) compared to upward changes (range 102–145 ms) (rm ANOVA; *F*(1,11) = 88.9, *p* < 0.0001). Note that the increase of latency with decrease of rate implies a longer latency with a longer sweep duration for the same frequency step magnitude.

The P2 peak latency followed the N1 peak by approximately 80–90 ms for each frequency step and rate. To investigate whether the P2 latency was influenced by the stimulus parameters in a different way than the N1 latency, the latency difference between P2 and N1 was analyzed. This latency difference was not significantly affected by magnitude, rate, or direction of the frequency change (rm ANOVA; *p* > 0.2).

### Direction Preference

**Fig. 6:**
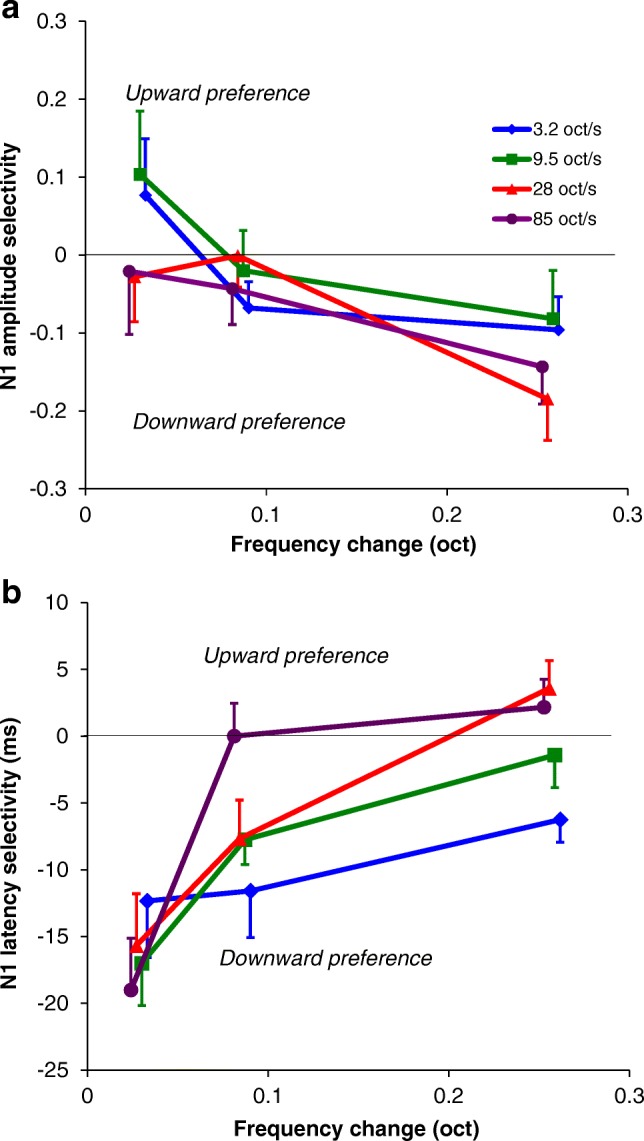
**a** N1 amplitude direction selectivity, expressed by DSI, as a function of frequency change (*n* = 12). **b** N1 latency direction selectivity, expressed by latency difference, as a function of frequency change (*n* = 12). Positive values represent an upward preference (larger amplitude or shorter latency) and negative values a downward preference. Purple lines indicate the sweeps of 85 oct/s, red lines of 28 oct/s, green lines of 9.5 oct/s, and blue lines of 3.2 oct/s. Error bars indicate standard errors of the mean. Random jitter has been added to avoid overlapping data points

Since both the amplitude and the latency of the N1 peak depended on the direction of the frequency change, as indicated by the rm ANOVA statistics; the direction preference was further analyzed for amplitude (Fig. 6a) and latency (Fig. 6b). The direction selectivity as computed for amplitude (DSI) was around 0 for the smaller magnitudes of change; a downward preference appeared for the largest magnitude applied, in particular for the faster sweeps. For individual stimulus conditions, downward preferences were not significant (one-sample *t* test, Bonferroni corrected, *p* > 0.05).

For the N1 latency, there is a downward preference for most conditions (Fig. 6b), also reflected by Fig. 5. Where DSI is larger for larger magnitudes, the latency difference is larger for the smaller frequency steps. The smallest frequency change of 0.028 oct displayed a significant downward preference for the higher rates (paired samples *t* test, Bonferroni corrected 9.5 oct/s *t*(11) = 5.36, *p* = 0.002; 28 oct/s *t*(11) = 4.05, *p* = 0.024; 85 oct/s *t*(11) = 4.91, *p* = 0.006). Significantly shorter latencies for downward changes were found at 0.086 oct and 9.5 oct/s (*t*(11) = 4.16, *p* = 0.024) and at 0.257 oct and 3.2 oct/s (*t*(11) = 3.69, *p* = 0.047).

## Discussion

This study’s objective was to investigate the effect of magnitude, rate, and direction of frequency changes on ACCs evoked in normal-hearing human subjects. Our results show that both amplitude and latency of the N1 peak are strongly affected by magnitude, rate, and direction of the frequency change, i.e., larger and earlier N1 peaks can be evoked by increasing the magnitude and/or rate of the frequency change and with downward changes. The P2 component showed a similar effect although only its amplitude was influenced by magnitude and, to a lesser extent, rate of the frequency change while direction had no effect on this peak. Recent literature has aimed at correlations of ACC measures with psychophysical outcomes in order to develop a clinical tool. It is therefore important to know, and relevant for researchers investigating the ACC, that the ACC is affected not only by magnitude but also, secondarily, by rate and direction.

### Effects of Magnitude and Rate of the Frequency Change on ACC Amplitude and Latency

The increase of ACC N1 and P2 amplitudes and the decrease of N1 latency with increase of frequency change are in line with earlier studies (McCandless and Rose 1970; Martin and Boothroyd 2000; Harris et al. 2008; Pratt et al. 2009; He et al. 2012). Harris et al. (2008) and He et al. (2012) found that a frequency change of around 1 % of the base frequency was required to evoke a discernable cortical response in young normal-hearing volunteers. In line with their results, all subjects in our study displayed a clear response with the smallest frequency change of 0.028 oct which corresponds with approximately 2 %.

In addition to the effect of magnitude, our results demonstrated that frequency change rate has a positive effect on N1 amplitude and, to a lesser extent, on P2 amplitude. In correspondence with the effect on amplitude, we found shorter N1 latencies with higher velocities.

This illustrates that the evoked cortical response is dependent on both frequency magnitude and rate, which can be explained as follows. The frequency change consists of an FM sweep and the target tone. Thus, the evoked response consists of the sum of the responses to the FM sweep and to the target tone. An individual cortical neuron may be sensitive to both components or to either of the two (Heil et al. 1992; Shamma et al. 1993; Kowalski et al. 1995; Nelken and Versnel 2000). In primary areas, the response to a pure tone is relatively strong; in nonprimary areas, the response to the FM sweep is relatively strong (Tian and Rauschecker 2004). Cortical neurons respond to the pure tone and FM sweep as long as these fall within the excitatory frequency response area. The number of neurons responding to the FM sweep increases with increase of the sweep magnitude, since the sweep passes through more excitatory frequency areas. Consequently, the evoked response increases with magnitude as indeed we found. The response of an individual neuron to the FM sweep may depend on direction and rate (Heil et al. 1992; Kowalski et al. 1995; Nelken and Versnel 2000; Tian and Rauschecker 2004; Trujillo et al. 2011), while the response to the pure-tone component will not depend on those parameters. Direction and rate selectivities vary widely across the auditory cortical areas; thus, some neurons respond to up sweeps rather than to down sweeps, and some neurons respond to slow sweeps rather than to fast sweeps. The summed evoked response to the FM sweep may actually not vary greatly with direction or rate if neurons with direction and rate selectivities are symmetrically and uniformly distributed. However, dependence on rate can be explained as follows. First, with faster sweeps, the neurons responding to the sweep respond more synchronously. Second, in case of short sweeps, the response to the target tone (the 300-ms tone following the FM sweep, as described in “Methods”) will follow the response to the FM sweep quite shortly (within 10 ms). This results in a larger total evoked response, whereas in case of a long sweep (in particular 81 ms; see Fig. 1), the responses to FM sweep and target tone will not overlap. Indeed, at the largest step, the responses were substantially smaller for the slowest sweep (where the sweep was longest) than for the other sweeps (Fig. 3). The decrease of latency with increase of rate can be further explained by assuming the response reaches its maximum during the sweep. For instance, considering the ACCs with a decrease of rate for the largest frequency step (0.26 oct), we see an increase of latency of about 20 ms from fastest to slowest sweep while the duration of the sweep increases by 78 ms (81–3 ms), suggesting the response peaks after a quarter of the sweep. For shorter steps, the response may peak towards the end of the sweep (latency increase of 8 ms while sweep increases by 9 ms).

### Comparison to ACCs Reported in Literature

Compared to the current study, Dimitrijevic et al. (2008), Harris et al. (2008), and He et al. (2012) all reported considerably smaller N1–P2 amplitudes varying between 2.5 and 5 μV in response to frequency increases (magnitudes varying 8 to 50 %) within pure tones. The largest frequency change magnitude in our study, which is 0.257 oct (approximately 20 %), generated considerable larger N1 amplitudes between 8.5 and 10.7 μV. This difference in amplitude between studies could occur due to use of different stimuli. Our results demonstrate that cortical potential recordings in response to frequency changes are highly stimulus dependent. In the current study, the first stimulus component before the change has a duration of approximately 3 s. This duration was based on our pilot data obtained in 3 subjects, which indicated that prolonging this component duration from 1 to 3 s generates approximately 30–50 % larger amplitudes. The stimuli used by Dimitrijevic et al. (2008) and He et al. (2012) had a duration of respectively 400 ms and 1300 ms before the change occurred. The enhanced neural responsiveness with a longer stimulus duration before the change might be facilitated due to increased firing ability of separate neurons. Wang et al. (2005) demonstrated that activity of single neurons within the monkey auditory cortex decreases with time during presentation of a 5000-ms constant tone, in particular in response to nonpreferred frequencies. A longer duration of the pure tone before the change could therefore facilitate low activity and thus a generally increased ability to respond to a new stimulus. Harris et al. (2008) reported an N1 amplitude of only 2.5 μV in response to an 8 % frequency increase, which is much smaller than we observed (Figs. 3 and 4). The duration of the reference tone was similar in both studies (~ 3 s); however, Harris et al. (2008) presented continuous stimuli, whereas we applied silent intervals of 200 ms allowing dishabituation and more neural recovery (Martin et al. 2007; Näätänen and Picton 1987), which might explain the larger amplitudes in the current study.

The N1 latencies we found (between 102 and 145 ms) are similar to values reported in the literature, including the strong dependence on magnitude of the change. Harris et al. (2008) reported a latency of approximately 132 ms in response to an 8 % frequency increase, He et al. (2012) reported an N1 latency of 110 ms in response to 20 % frequency changes, and Dimitrijevic et al. (2008) reported latencies of approximately 105 ms in response to a 50 % frequency increase. Indeed, these studies together showed consistently shorter latencies with larger changes. These studies did not report the change rate of their stimuli; therefore, minor differences between studies might occur due to the effects of both rate and varying durations of the first stimulus component. Brown et al. (2015) recorded ACCs in normal hearing subjects in response to changing vowel sounds with the second formant shifting an octave at 400 ms after onset. This study also reported amplitudes (N1–P2 amplitude 7 μV) and latencies (N1 latency approximately 120 ms) which agree with our values considering on the one hand the large change and on the other hand the short duration of the first component.

### Direction Preference

Previous studies using cortical evoked potentials have reported contrasting results with respect to the dependence on frequency change direction (Arlinger et al. 1976; Arlinger and Jerlvall 1979; Maiste and Picton 1989; Pratt et al. 2009). Maiste and Picton (1989) and Pratt et al. (2009) reported larger amplitudes for frequency increases than decreases in contrast to the studies by Arlinger et al. (1976) and Arlinger and Jerlvall (1979) reporting no difference in amplitude between upward or downward sweeps. Brown et al. (2015) used vowel transitions and did not find any differences in ACCs between increases and decreases of the formant frequency. In contrast to these studies, our study demonstrates a downward direction preference for the N1 peak, for the small changes expressed by shorter latencies and for large changes expressed by larger amplitudes. In the current study, we chose to use separate recordings in a randomized order to control for possible interactions between successive stimuli. Moreover, for direction selectivity analyses, we corrected for variance in amplitudes, rather than using absolute peak amplitudes. Inter-subject variance in ACC amplitudes could severely influence calculations of direction selectivity if individual peak amplitudes are averaged.

Taken together, the results of the abovementioned studies indicate that the existence of a general direction preference is weak at most. Various psychophysical studies, which examined detection ability in human speech and/or spectrotemporal modulations, confirm this notion (Schouten and Pols 1985; Schouten 1986; Dooley and Moore 1988; Chi et al. 1999; Gordon and Poeppel 2002; Luo et al. 2007). It implies that in human auditory cortex up- and downward directions, selective neurons are roughly equally distributed. Preference may be dependent on specific stimulus conditions as suggested by Nelken and Versnel (2000) and as indicated by the current study.

### Clinical Implications

A literature review by Kim (2015) concluded that the ACC as an objective measurement shows reasonable agreement with psychophysical measures and can be reliably recorded in normal-hearing subjects, hearing impaired patients, and cochlear implant users. The ACC might therefore hold clinical value as an objective tool in hearing impairment. When investigating clinical applications, one should be aware the ACC is affected not only by magnitude but also by rate and direction of the change. In order to obtain the clearest response, a large and fast FM sweep is the appropriate stimulus to be used.

## Abbreviations

ACC: Acoustic change complex
FM: Frequency modulation
oct: Octave
RM: Repeated measures
